# Phylogenetic Analysis of Spread of Hepatitis C Virus Identified during HIV Outbreak Investigation, Unnao, India

**DOI:** 10.3201/eid2804.211845

**Published:** 2022-04

**Authors:** Arati Mane, Sunitha Manjari Kasibhatla, Pallavi Vidhate, Vandana Saxena, Sandip Patil, Amrita Rao, Amit Nirmalkar, Urmila Kulkarni-Kale, Samiran Panda

**Affiliations:** Indian Council of Medical Research–National AIDS Research Institute, Pune, India (A. Mane, P. Vidhate, V. Saxena, S. Patil, A. Rao, A, Nirmalkar, S. Panda);; Savitribai Phule Pune University Bioinformatics Centre, Pune (S.M. Kasibhatla, U. Kulkarni-Kale);; Centre for Development of Advanced Computing High Performance Computing–Medical and Bioinformatics Applications Group, Pune (S.M. Kasibhatla);; Indian Council of Medical Research, New Delhi, India (S. Panda)

**Keywords:** hepatitis C, viruses, HIV/AIDS, dual outbreak, general community, NS5B, core sequencing, phylogenetic analysis, Unnao, India

## Abstract

An HIV outbreak investigation during 2017–2018 in Unnao District, Uttar Pradesh, India, unearthed high prevalence of hepatitis C virus (HCV) antibodies among the study participants. We investigated these HCV infections by analyzing NS5B and core regions. We observed no correlation between HIV–HCV viral loads and clustering of HCV sequences, regardless of HIV serostatus. All HCV isolates belonged to genotype 3a. Monophyletic clustering of isolates in NS5B phylogeny indicates emergence of the outbreak from a single isolate or its closely related descendants. The nucleotide substitution rate for NS5B was 6 × 10^−3^ and for core was 2 × 10^−3^ substitutions/site/year. Estimated time to most recent common ancestor of these isolates was 2012, aligning with the timeline of this outbreak, which might be attributable to unsafe injection practices while seeking healthcare. HIV–HCV co-infection underlines the need for integrated testing, surveillance, strengthening of healthcare systems, community empowerment, and molecular analyses as pragmatic public health tools.

Hepatitis C virus (HCV) infection is a major public health concern worldwide and recognized as the leading cause of chronic liver disease, cirrhosis, and hepatocellular carcinoma (HCC). India, Pakistan, Georgia, and many countries in the western world have recorded high HCV prevalence among persons who inject drugs (PWID) (*1*–*4*). In addition, unsafe injection practices in therapeutic settings have been responsible for spread of HCV infection in the general population (*5*,*6*). HCV shares the same routes of transmission as HIV, although heterosexual sex is a less efficient one. Owing to the shared routes of transmission, about one third of persons living with HIV have been estimated to be coinfected with HCV in the United States, Europe, and some developing countries (*7*,*8*).

Increased detection of HIV among the attendees of the Integrated Counselling and Testing Center, located in the district hospital of Unnao, in the northern state of Uttar Pradesh, India, was reported in July 2017. A case–control investigation was initiated while 2 patients from Premganj Township of Bangarmau Block, Unnao, raised concerns that a local doctor (frequented mostly by persons from lower socioeconomic status) had been using a single syringe and needle to inject different persons. That investigation indicated that unsafe injecting practices in therapeutic settings were associated with HIV transmission and identified clustering around a monophyletic HIV-1 clade C. Serologic analyses revealed very high prevalence of antibodies to HCV among HIV-infected persons (85%) and controls (56%), suggesting a concerning level of spread of HCV infection in the study community, which was a serendipitous finding (*5*). Detection and molecular analysis of community HCV outbreaks is important because they can inform targeted public health interventions to interrupt HCV transmission. These initiatives are even more imperative as India aims to achieve the Sustainable Development Goal 3.3, which seeks to end viral hepatitis by 2030, as articulated in the National Health Policy (*9*). In this article, we describe the findings of molecular and phylogenetic analysis of HCV infections discovered during the HIV outbreak investigation in Unnao, India.

## Methods

### Ethics Statement

We obtained approval for the investigation from the Ethics Committee of the Indian Council of Medical Research–National AIDS Research Institute (Pune, India). All study participants provided written informed consent.

### Clinical Specimens and Data

We conducted molecular and phylogenetic analyses for 98 HCV seroreactive participants, including 70 with HCV and 28 with HIV–HCV co-infection. We enrolled these participants as part of our previous case–control investigation, which was undertaken to identify the factors associated with increased detection of HIV in Unnao District Hospital, Unnao, India, during September–December 2018, details of which are published elsewhere (*5*). In brief, case-patients (n = 33) were persons found to be HIV seroreactive during a 6-month period (November 2017–April 2018) from 3 locations (Premganj, Karimuddinpur, and Chakmeerapur) in the Bangarmau Block of Unnao District. Controls (n = 125) were persons who lived in the same geographic locale as case-patients and tested HIV serononreactive either in health camps or at the Integrated Counselling and Testing Centers where the cases were detected. Eighty-five percent (28/33) of HIV-positive and 56% (70/125) of HIV-negative study participants were HCV seroreactive. We used the HCV seroreactive specimens stored at −80°C for the molecular analysis for HCV infection, along with corresponding metadata pertaining to sociodemographic and laboratory data. 

#### Viral Load Estimation

We performed HCV viral load testing by using the Xpert HCV viral load assay (Cepheid, https://www.cepheid.com). In brief, we placed 1 mL serum into the Xpert cartridge, scanned it, and loaded it into the GeneXpert IV instrument. We recorded the results according to the manufacturer’s instructions.

We estimated HIV-1 viral load by using the Abbott Real Time HIV-1 assay (Abbot https://www.abbott.com), which uses reverse transcription PCR (RT-PCR) with homogenous detection of real-time fluorescence. We carried out automated sample preparation on the m2000sp instrument for RNA isolation by using magnetic microparticle technology and dispensed it to a PCR tray along with the amplification reagents. We then transferred the PCR tray to the Abbott m2000rt instrument for amplification and real-time detection according to the manufacturer’s instructions.

#### HCV RNA Extraction, NS5B and Core Gene Amplification, and Sequencing

We extracted viral RNA by using the NucliSens EasyMag total nucleic acid extraction system (bioMérieux, https://www.biomerieux.com). We amplified the extracted RNA for the core and NS5B region of HCV. We amplified the core region with outer sense primer 5′-ACTGCCTGATAGGGTGCT TGC-3′; outer antisense primer 5′-ATGTACCCCATGAGGTCGGC-3′; inner sense primer 5′-AGGTCTCGTAGACCGTGCA-3′; and inner antisense primer 5′-CATGTGAGGGTATCGATGAC-3′ (*10*). We amplified the NS5B region with outer sense primer 5′-CNTAYGGITTCCARTACTCICC-3′, antisense primer 5′-GAGGARCAIGATGTTATIARCTC-3′, inner sense primer 5′-TATGAYACCCGCTGYTTTGACTC-3′ and inner antisense primer 5′-GCNGARTAYCTVGTCATAGCCTC-3′ (*11*). We used the Onestep RT-PCR kit (QIAGEN, https://www.qiagen.com) for cDNA synthesis. We carried out the outer PCR in 25 µL reaction containing 4 µL 5× PCR buffer, 1 µL 2.0 mmol/L dNTP, 4.5 µL RNase-free water, 2 µL each sense and antisense primers, 1 µL RT-PCR enzyme mix, 0.5 µL RNase out (Invitrogen, https://www.thermofisher.com), and 10 µL extracted RNA (treated with heat at 65°C for 30 s for core and 42°C for 5 min for NS5B) with PCR conditions: 50°C for 35 min; 95°C for 15 min; 95°C for 20 s, 55°C for 45 s, 72°C for 2 min, 35 cycles; and 72°C for 10 min. We carried out the inner PCR in 25 µL reaction containing 2.5 µL 10× PCR buffer, 1 µL 2.5 mmol/L dNTP, 11 µL water, 1 µL each sense and antisense primers, 0.5 µL Taq enzyme, and 5 µL template cDNA with PCR conditions: 95°C for 1 min, 95°C for 15 s, 56°C for 45 sec, 72°C for 1 min, 30 cycles; and 72°C for 5 min. We verified the PCR products by gel electrophoresis. We sequenced all amplified products on the Applied Biosystems 3130XL genetic analyzer (Applied Biosystems/ThermoFisher, https://www.thermofisher.com). We then performed sequence assembly and base-calling with SeqScape 2.7 software (ThermoFisher), using the FASTA files generated for further analysis. We submitted nucleotide sequences to GenBank (accession nos. MW675966–MW676030 for the core gene and MW675899–MW675965 for the NS5B gene).

#### HCV Genotyping and Phylogenetic Analysis

We genotyped partial sequences of core and NS5B genes of HCV samples from persons with HIV–HCV coinfection (labeled as i) and HCV monoinfection (labeled as ic) by using GenomeDetective (https://www.genomedetective.com/app/typingtool/hcv) and HCV-Blast (*12*). We performed multiple sequence alignments by using the prototype sequence of H77 isolate (GenBank accession no. NC_038882) belonging to genotype 1, using MAFFT (*13*). We determined the exactly identical sequences by using the CD-HIT web server (*14*). We then extracted core and NS5B genes belonging to India and global HCV isolates from GenBank (*15*) by using blastn (*16*). We carried out recombination detection by using RDP4 (*17*) and then reconstructed phylogenetic trees for core and NS5B sequences by using the maximum-likelihood method as implemented in IQTREE (*18*). We used partial sequences of core and NS5B genes belonging to other India and global isolates, which clustered with Unnao samples for evolutionary analysis. We used BEAST version 1.10.4 to determine time to most recent common ancestor (tMRCA) (*19*). 

We used the generalized time reversible model as a nucleotide substitution model with relaxed clock and log-normal distribution and coalescent constant growth as demographic model for tMRCA calculation. We carried out Markov Chain Monte Carlo simulations for 100 million steps and sampled every 10,000 steps. We used Tracer 1.6 (https://bioweb.pasteur.fr/packages/pack@Tracer@v1.6) for assessing convergence and iTOL (https://itol.embl.de) and FigTree (https://github.com/rambaut/figtree) for visualization of phylogenetic trees. To generate information on drug-resistant mutation substitutions observed in NS5B protein sequences, we mapped to sofosbuvir and ribavirin drug-resistant variants (http://hcv.geno2pheno.org).

### Statistical Analysis

We compared the means between 2 samples by using an independent *t* test. We used Pearson’s correlation analysis to determine whether the values of 2 variables were associated. We considered a probability level lower than the conventional 5% (p<0.05) as statistically significant. We assessed the association between dependent variable and cofactors by using Pearson's χ^2^ or Fisher exact tests as appropriate.

## Results

### Participant Profile

We profiled persons with HCV monoinfection (n = 70) and those with HIV–HCV co-infection (n = 28) (Table 1); sex, area of residence, occupation, and sexual risk distribution were not significantly different between the 2 groups. We observed statistically significant differences between the 2 groups with regards to age and receipt of injection during treatment-seeking within the last 5 years.

**Table 1:** Characteristics of anti-HCV positive persons identified during HIV outbreak investigation, by HIV serostatus, Unnao, India*

Characteristic	HIV–HCV co-infection	HCV monoinfection	p value
Total no. persons	28 (100)	70 (100)	
Mean age, y	50	38	0.044
Sex			
M	09 (32.1)	33 (47.1)	0.175
F	19 (67.9)	37 (52.9)	
Area of residence			
Chakmeerapur	12 (42.9)	32 (45.7)	0.957
Kirvidyapur	1 (3.6)	2 (2.9)	
Premganj	15 (53.6)	36 (51.4)	
Occupation			
Unemployed	17 (60.7)	29 (41.4)	0.147
Farmer	5 (17.9)	12 (17.1)	
Nonagricultural	6 (21.4)	29 (41.4)	
Ever had sex with female casual partner (as reported by male participants)†			
Yes	1 (14.3)	2 (6.1)	0.453
No	6 (85.7)	31 (93.9)	
Ever had sex with male casual partner (as reported by female participants)†			
Yes	1 (5.26)	0	0.345
No	18 (94.74)	36 (100)	
Condom use during last sex†			
Yes	3 (12.5)	11 (17.2)	0.750
No	21 (87.5)	53 (82.8)	
Intravenous injection in therapeutic setting in past 5 years			
Yes	27 (96.4)	47 (67.1)	0.002
No	1 (3.6)	23 (32.86)	
Intramuscular injection in therapeutic setting in past 5 years			
Yes	27 (96.4)	45 (64.3)	0.001
No	1 (3.6)	25 (35.7)	
Way syringe and needle used while receiving intramuscular injection in past 5 years†			
Injected by used syringe and needle	9 (32.1)	3 (4.3)	0.0001
Injected by new syringe and needle	13 (46.4)	60 (85.7)	

*Values are no. (%) except as indicated. HCV, hepatitis C virus. †Because of nonresponse from some participants, numbers may not sum to total.

We compared the characteristics of HCV reactive (n = 98) and nonreactive (n = 60) persons and observed that a statistically significant higher proportion of HCV reactive persons reported being exposed to used syringes and needles while seeking treatment and were HIV seropositive compared with HCV nonreactive persons. We did not observe significant differences with regards to age, sex, area of residence, occupation, and sexual risk distribution between the 2 groups (Appendix Table). None of the study participants reported ever injecting drugs for nonmedicinal or recreational purposes.

#### Association between HCV and HIV Viral Load

We tested all HCV antibody-positive specimens for HCV viral load. Samples collected from 2 participants with HIV–HCV co-infection (identified by presence of antibody) and 3 with HCV monoinfection (seroreactive) had insufficient volume. Thus, we used specimens from 26 participants with HIV–HCV co-infection and 67 participants with HCV monoinfection for viral load testing. Eighty-eight percent (23/26) of specimens from HIV–HCV coinfected persons and 73% (49/67) specimens from HCV monoinfected participants had detectable HCV RNA (chronic infection). HCV RNA load in participants with HIV–HCV co-infection was (mean + SD log IU/mL) 5.93 + 0.91 and HCV monoinfection was 5.46 + 1.09; the difference was not statistically significant (p = 0.07). HCV RNA load did not differ significantly in HIV-positive persons with detectable (6.06 + 1.15) and undetectable (5.85 + 0.76) viral load (p = 0.65) (Table 2). We did not observe any correlation between HIV and HCV viral load (r = 0.0567; p = 0.88).

**Table 2:** Viral load among HCV antibody–positive persons identified during HIV outbreak investigation, by HIV serostatus, Unnao, India*

Category	Viral load, log IU/mL, + SD	p value
Persons with HCV monoinfection	5.46 + 1.09	0.07†
Persons with HIV–HCV co-infection	5.93 + 0.91	
Persons with undetectable HIV-1 viral load	5.85 ± 0.76	0.65‡
Persons with detectable HIV-1 viral load	6.06 + 1.15	

*HCV, hepatitis C virus. †For comparison between HCV monoinfection and HIV–HCV co-infection. ‡For comparison between undetectable and detectable HIV-1 viral load.

#### Genetic Variability in the HCV Core and NS5B Genes and HCV genotypes

We obtained a total of 67 (23 with HIV–HCV coinfection and 44 with HCV monoinfection) and 65 (22 with HIV–HCV coinfection and 43 with HCV monoinfection) sequences belonging to core and NS5B regions. The partial core sequence mapped to 394–722 (329 bases) and NS5B gene mapped to 8,196–8,647 (452 bases) of H77 prototype. We observed the extent of sequence similarities in the core HIV–HCV coinfection was 98.15%, in HCV monoinfection was 96.9%, and in combined datasets of the sequence similiarity was 96.6%. Similarly, for the NS5B sequence similarities in HIV–HCV coinfection was 98.9%, HCV monoinfection was 98.03%, and in combined datasets of the sequence similarity was 97.36%. All the isolates from Unnao belonged to genotype 3a regardless of HIV co-infection status. No recombination was observed in the regions of NS5B and core genes sequenced.

With reference to the H77 prototype, we obseved a total of 71 nucleotide and 12 amino acid substitutions in the core protein, whereas the NS5B protein showed 70 nucleotide and 43 amino acid substitutions in Unnao isolates. Of the 12 amino acid substitutions in the core protein, 6 (N16I, L36V, R70Q, P71S, T75S, and T110N) were part of highly variable sites in other India isolates. Of the 43 NS5B substitutions, 6 were associated with resistance to the known drugs ribavirin and sofosbuvir. The substitutions Q309R (observed in all Unnao isolates) and R345S (observed in 4 isolates in the HCV monoinfection group) are known to be associated with resistance to ribavirin. We also observed the substitutions A207M, S218F, C289F, and A333R (associated with sofosbuvir resistance) in all isolates.

#### HCV Phylogenetic and Evolutionary Analysis

**Figure 1:**
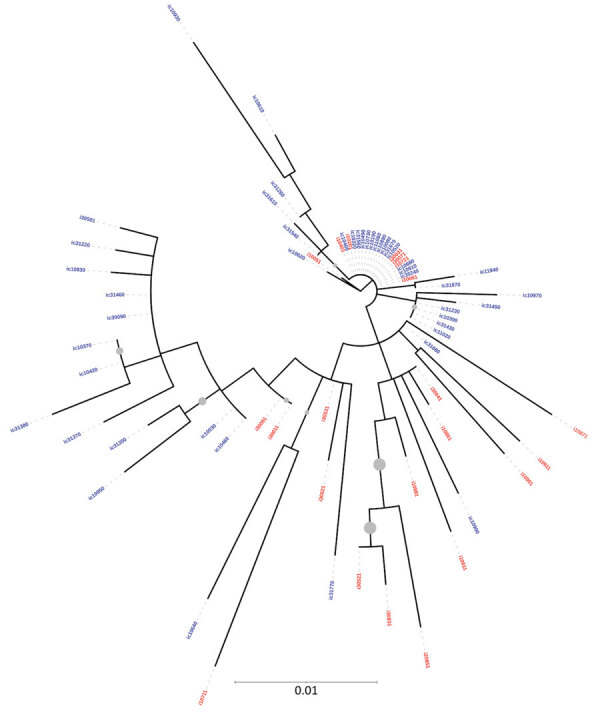
Maximum-likelihood phylogenetic tree derived using hepatitis C virus (HCV) NS5B gene sequences of isolates from anti-HCV positive persons identified during HIV outbreak investigation, Unnao, India. Grey circles indicate nodes with >70% bootstrap support. Red indicates samples with HIV–HCV co-infection; blue indicates samples with HCV monoinfection. i, HIV–HCV co-infection; ic, HCV monoinfection.

**Figure 2:**
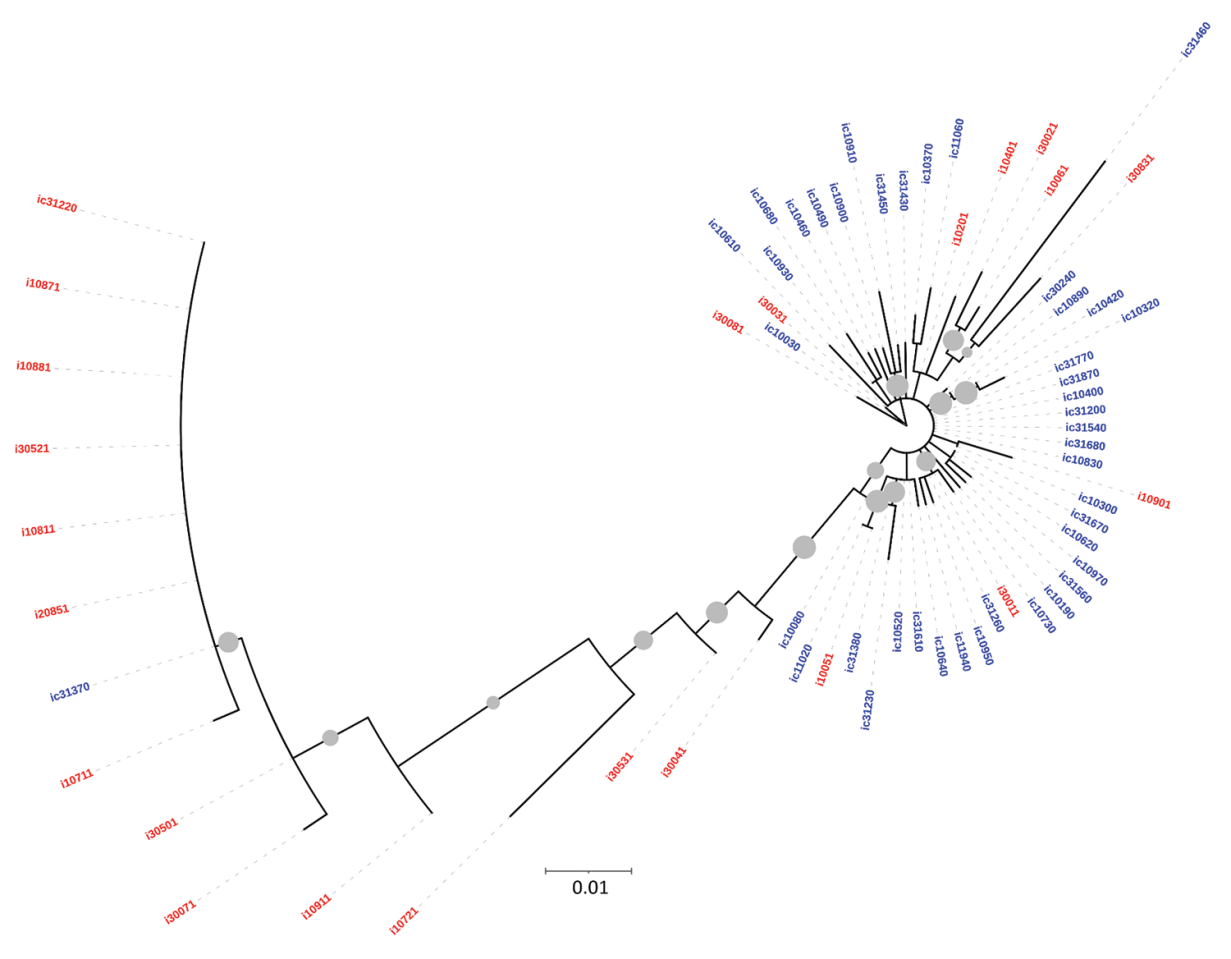
Maximum-likelihood phylogenetic tree derived using hepatitis C virus (HCV) core gene sequences of isolates from anti-HCV positive persons identified during HIV outbreak investigation, Unnao, India. Grey circles indicate nodes with >70% bootstrap support. Red indicates samples with HIV–HCV coinfection; blue indicates samples with HCV monoinfection. i, HIV–HCV co-infection; ic, HCV monoinfection.

The phylogenetic trees derived using partial sequences of NS5B and core genes of the Unnao isolates showed clustering of isolates with HIV–HCV co-infection and HCV monoinfection, indicating lack of distinct clusters pertaining to HIV status (Figures 1, 2). In the phylogenetic tree derived for NS5B gene using global sequences that include India HCV isolates from other studies as well as the Unnao isolates, Unnao isolates were observed to form a monophyletic cluster (Appendix Figure 1). The closest branch joining the Unnao isolates includes 1 India isolate (GenBank accession no. GQ275355, isolated during 2003), 3 isolates from the United States (GenBank accession nos. DQ430819–20, isolated during 2000, and AY956467, isolated in 2002), and 2 isolates from the United Kingdom (GenBank accession nos. GQ356207 and GQ356217, isolated during 2006). In the phylogenetic analysis using the core gene, Unnao isolates were part of 3 clusters. Most Unnao isolates (56 of the total 65) formed a distinct cluster. The second cluster included 1 Unnao isolate ic31460 and 5 HCV isolates (GenBank accession nos. MT953835 and MT953776, isolated during 2019 from India, and GenBank accession nos. KY620638, KY620807, and KY620806, isolated during 2014 [country of isolation is not available]). The third cluster included Unnao isolates i10711, i30501, i10911, i30071, i20851, i10811, ic31220, and ic31370 and other India isolates (GenBank accession nos. MN697780, isolated during 2016, and MN697854 and MN697827, isolated during 2017) from a previous study (*20*) (Appendix Figure 2).

**Figure 3:**
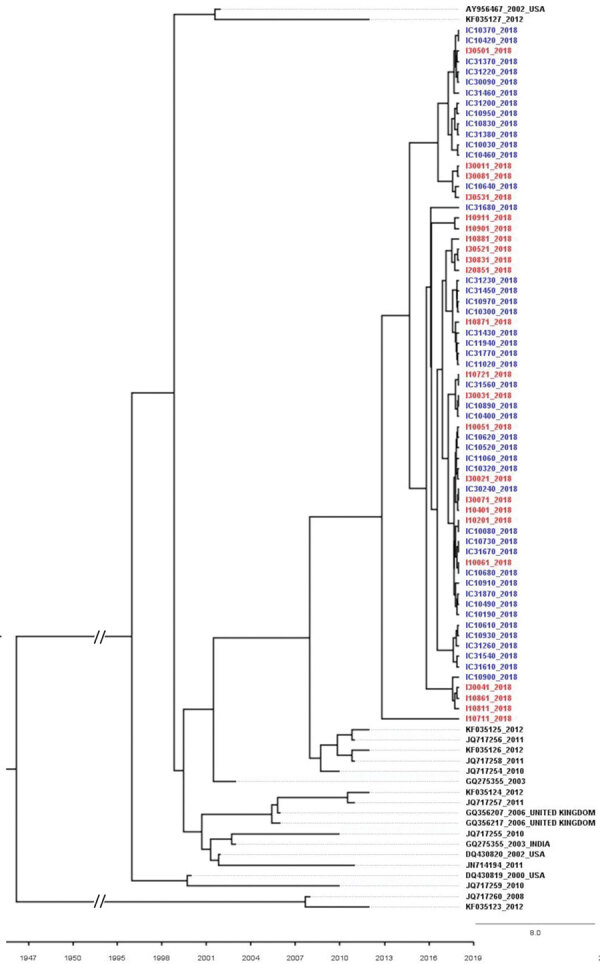
Time to most recent common ancestor estimated using hepatitis C virus (HCV) NS5B gene sequences of isolates from HCV antibody–positive persons identified during HIV outbreak investigation, Unnao, India. Red indicates samples with HIV–HCV coinfection; blue indicates samples with HCV monoinfection. Mean estimated time to most recent common ancestor of HCV isolates with the respective 95% highest posterior density interval is 2012 (2008–2014), with posterior probability value of 1. GenBank accession numbers are provided for reference sequences. Scale bar indicates branch lengths. I, HIV–HCV co-infection; IC, HCV monoinfection.

We estimated tMRCA for Unnao isolates by using the partial sequences of NS5B genes of HIV–HCV co-infection and HCV monoinfection, together with the India and global isolates that are observed in close proximity in the phylogenetic tree. tMRCA for Unnao was observed to be 2012 (Figure 3). The nucleotide substitution rate of partial sequences of the core gene belonging to Unnao and global isolates is 2 × 10^−3^ substitutions/site/year (95% highest posterior density interval 1.16–3.54 × 10^−3^) and for the NS5B gene (that clustered with Unnao isolates) is 6 × 10^−3^ substitutions/site/year (95% highest posterior density interval 1.28–8.46 × 10^−3^).

## Discussion

We describe the investigation of a HIV–HCV dual outbreak in a general community setting from India by means of sequencing of partial regions of 2 HCV genes; namely, NS5B (nonstructural) and core (structural). The NS5B is relatively conserved and used as a marker for genotyping (*21*). The isolates sequenced from participants with HCV monoinfection and co-infection (HIV–HCV) were characterized by using phylogenetic analysis to understand the circulating genotypes, time of introduction of HCV in the community, and the functional significance of substitutions, if any.

Phylogenetic analysis revealed the presence of genotype 3a in all samples. This genotype is the most commonly reported from the state of Uttar Pradesh and the neighboring regions (*2*,*22*,*23*). All HCV sequences from Unnao shared high sequence similarity (>96%) regardless of HIV infection status. The clustering pattern observed in the phylogenetic tree derived from NS5B suggests monophyly and thereby indicates that the Unnao isolates are descendants of 1 (or many) closely related 3a isolates that are circulating in the community. Similarly, the estimated nucleotide substitution rates reported in our study are not only in agreement with previous reports (*24*,*25*) but also corroborate with the timeline of the HIV outbreak reported. Thus, the phylogenetic clustering supports the observation pertaining to the use of unsterile injection equipment as a potential route of entry of HCV in the study setting (*5*). The phylogenetic tree of core sequences from Unnao showed that 1 of the 3 observed clusters consisting of 8 sequences (6 i and 2 ic) group with isolates reported in a previous study describing HCV diversity among HIV-infected persons (*20*). However, clustering proximity of Unnao isolates with isolates from recreational drug users (based on core region) is insufficient (*21*) and therefore the use of recreational drug abuse can be ruled out in the Unnao outbreak (Appendix Figure 2). None of the study participants reported injecting drugs for nonmedicinal or recreational purposes. Thus, the exceptionally high rate of HCV infection in Unnao might be explained by exposure of the local community (mostly agrarian and belonging to lower socioeconomic status) to unsafe injection practices during healthcare seeking; an assertion supported by the observation of 2 patients, which prompted the case–control study described previously and earlier reports documenting reuse of injection equipment in nearby rural and urban settings (*5*,*26*).

HIV is reported to accelerate the progression of liver disease in HCV-infected persons because HCV replication increases in the presence of HIV, resulting in elevated HCV RNA levels (*7*,*25*). We did not observe correlation between HIV and HCV RNA load, nor was any specific clustering of HCV sequences based on HIV serostatus noted. Thus, it appears that the influence of duration of HIV infection was not sufficiently strong and probably succeeded (rather than preceded) the highly evolved or adapted HCV that circulated in defined clusters.

We observed certain mutations associated with ribavirin and sofosbuvir resistance; these drugs form the main component of HCV treatment regimens in India (*9*). The low detection rate of resistance mutations in the isolates corroborates with the HCV treatment–naive status of the community in concern. Knowledge of baseline mutations is important for predicting potential response to antiviral therapy and will help to determine local policies for HCV treatment.

The first limitation of our study is that it was not primarily designed as an HCV outbreak investigation and hence was restricted in terms of the samples tested for HCV phylogeny. The nature of the investigation did not enable us to link HCV infection to any specific source. HCV RNA testing was performed after the detection of the HIV outbreak in Unnao. Hence, we could not define the time between infection and the onset of viremia. A similar outbreak investigation in the Roka rural commune in Cambodia showed that concurrent spread of HIV and HCV was linked with unsafe injecting practices in therapeutic settings (*6*).

The ongoing HCV epidemic we uncovered in our investigation highlights the need for HCV surveillance in the area and in neighboring districts. Furthermore, after our investigation, an antiretroviral treatment center was established at the Bangarmau Community health center, the coordination site for the case–control investigation described previously, to ensure access of local residents to testing, treatment, and follow-up services.

In conclusion, characterization of NS5B and core sequences with HCV monoinfection and HIV–HCV co-infection using phylogenetic analysis not only substantiates our previous results (*5*) but also indicates a potential HCV outbreak in the community from a single isolate or its closely related descendants in Unnao, India. The HIV–HCV dual epidemic in a general community setting highlights the need for systematic surveillance and integrated approach combining hepatitis testing along with HIV for early detection and timely interventions to arrest transmission. We reiterate the need for adhering to good infection prevention and control practices, deploying single-use injection equipment (preferably autodisabled ones), strengthening healthcare delivery systems, and empowering the community to reduce the further spread of HIV and HCV. Molecular analyses of all HCV outbreaks is warranted to decipher the circulating genotypes, genetic diversity, and transmission dynamics. These findings in turn will provide valuable inputs to the National Viral Hepatitis Control Program in planning targeted interventions for mitigating the spread of HCV in India.

Appendix Additional information about phylogenetic analysis of spread of hepatitis C virus identified during HIV outbreak investigation, Unnao, India.
