# Supplementary material for: Phylogenetic Analysis of Spread of Hepatitis C Virus Identified during HIV Outbreak Investigation, Unnao, India
Source: Emerg Infect Dis. 2022 Apr;28(4):725–33. doi: 10.3201/eid2804.211845 (PMC8962895; doi:10.3201/eid2804.211845)
Supplement: Appendix — Additional information about phylogenetic analysis of spread of hepatitis C virus identified during HIV outbreak investigation, Unnao, India. [file 21-1845-Techapp-s1.pdf]

Article DOI: https://doi.org/10.3201/eid2804.211845

# Phylogenetic Analysis of Spread of Hepatitis C Virus Identified during HIV Outbreak Investigation, Unnao, India

## Appendix

**Appendix Table.** Characteristics of anti-HCV reactive and nonreactive persons identified during HIV outbreak investigation, Unnao, India, by HIV serostatus*

| Characteristic | Anti-HCV reactive | Anti-HCV nonreactive | p-value |
|---|---|---|---|
| Total no. persons | 98 (100) | 60 (100) | |
| Mean age, y | 41 | 38 | 0.289 |
| Sex | | | |
| M | 42 (42.86) | 29 (48.33) | 0.515 |
| F | 56 (57.14) | 31 (51.67) | |
| Area of residence | | | |
| Chakmeerapur | 44 (44.90) | 26 (43.33) | 0.419 |
| Kirvidyapur | 03 (3.06) | 05 (8.33) | |
| Premganj | 51 (52.04) | 29 (48.33) | |
| Occupation | | | |
| Unemployed | 46 (46.94) | 19 (31.67) | 0.157 |
| Farmer | 17 (17.35) | 15 (25.00) | |
| Non-agricultural | 35 (35.71) | 26 (43.33) | |
| Ever had sex with female casual partner as reported by male participants† | | | |
| Yes | 03 (7.50) | 03 (10.71) | 0.684 |
| No | 37 (92.50) | 25 (89.29) | |
| Ever had sex with male casual partner as reported by female participants† | | | |
| Yes | 01 (1.82) | 0 | 1.000 |
| No | 54 (98.18) | 31 (100) | |
| Condom use during last sex† | | | |
| Yes | 14 (15.91) | 13 (24.07) | 0.273 |
| No | 74 (84.09) | 41 (75.93) | |
| Intravenous injection in last 5 years | | | |
| Yes | 74 (75.51) | 37 (62.71) | 0.104 |
| No | 24 (24.49) | 22 (37.29) | |
| Intramuscular injection in last 5 years | | | |
| Yes | 72 (73.47) | 38 (63.33) | 0.213 |
| No | 26 (26.53) | 22 (36.67) | |
| Syringe and needle used while receiving intramuscular injection in last 5 years† | | | |
| Injected by used syringe and needle | 12 (12.24) | 02 (3.33) | 0.046 |
| Injected by new syringe and needle | 73 (74.49) | 54 (90.0) | |
| HIV infection | | | |
| Seropositive | 28 (28.57) | 05 (8.33) | 0.002 |
| Seronegative | 70 (71.43) | 55 (91.67) | |

*Values are no. (%) except where indicated. HCV, hepatitis C virus.
†Because of nonresponse from some participants, numbers may not sum to total.

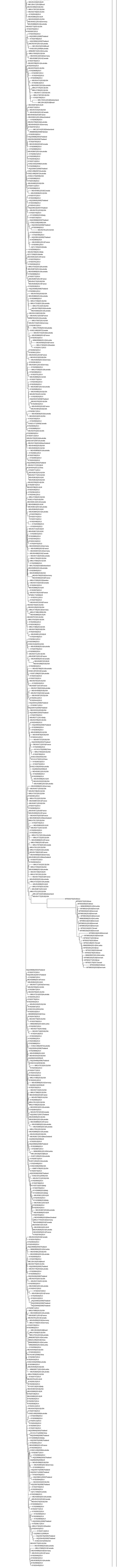

**Appendix Figure 1.** Phylogenetic tree of HCV global isolates derived using NS5B gene by maximum-likelihood method. Samples isolated from Unnao, India, sequenced in this study are shown in red for HIV/HCV co-infection cases and in blue for HCV monoinfection cases.

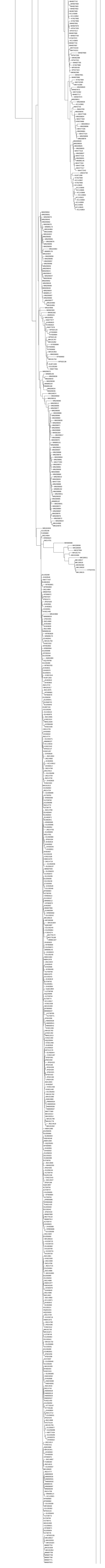

**Appendix Figure 2.** Phylogenetic tree of HCV global isolates derived using core gene by maximum-likelihood method. Samples isolated from Unnao, India, sequenced in this study are shown in red for HIV/HCV coinfection cases and in blue for HCV monoinfection cases.
